# P76 Use of delafloxacin for ciprofloxacin-resistant *Pseudomonas aeruginosa* foot osteomyelitis: a case report

**DOI:** 10.1093/jacamr/dlag102.082

**Published:** 2026-06-26

**Authors:** Beth Lillico, Anna Tilley, Rohit Bazaz

**Affiliations:** Manchester Local Care Organisation, Manchester, UK; Manchester University NHS Foundation Trust, Manchester, UK; Manchester University NHS Foundation Trust, Manchester, UK

## Abstract

**Background:**

Osteomyelitis associated with peripheral arterial disease poses a significant risk of limb loss, especially in frail older adults. There are limited published data on the efficacy of delafloxacin, a novel anionic fluoroquinolone, in osteomyelitis. Here, we describe its use in an elderly patient with foot osteomyelitis.

**Case study:**

An 89-year-old man was referred to podiatry with two ulcers on his right foot. His medical history included postural hypotension, previous stroke, temporal arteritis, diverticulitis, epilepsy, atrial fibrillation, peripheral arterial disease and active tobacco use. He was registered blind and severely frail (Rockwood Clinical Frailty Score of 7). On assessment, a right second dorsal interphalangeal joint ulcer was clinically concerning for osteomyelitis, with a positive probe-to-bone test, surrounding oedema and erythema. A lateral fifth metatarsal ulcer was classified as an unstageable pressure injury, not initially suspicious for osteomyelitis. An X ray showed no evidence of osteomyelitis around the second interphalangeal joint but demonstrated osteomyelitis of the fifth metatarsal head. Given his frailty and relative clinical stability, a shared decision was made with the patient’s GP not to pursue urgent vascular intervention, opting for conservative management with close podiatry monitoring. Obtaining an adequate sample to guide antimicrobial therapy was challenging due to an exposed collateral ligament obscuring the wound bed, therefore tissue sampling was deferred. Empirical oral doxycycline was commenced, with weekly wound reviews and district nursing support. Despite six weeks of doxycycline, the fifth metatarsal ulcer deteriorated, which led to an extension of doxycycline therapy. A repeat x-ray at eight weeks of doxycycline treatment showed increased bone loss of the fifth metatarsal head consistent with ongoing active osteomyelitis. The patient remained systemically well but developed doxycycline associated side effects after completing 12 weeks in total. At 12 weeks, discharge from the still deteriorating fifth metatarsal wound enabled a sample to be sent for culture, and this grew ciprofloxacin resistant *Pseudomonas aeruginosa*. A subsequent deep bone sequestrum sample was obtained which also grew ciprofloxacin-resistant *P. aeruginosa*. Both isolates were susceptible at increased exposure to piperacillin/tazobactam and ceftazidime. Delafloxacin sensitivity testing was performed on the bone sample Pseudomonas isolate, demonstrating a favourable MIC of 0.5 mg/L. Following multidisciplinary discussions involving podiatry, primary care, Infection specialists, the patient and his carers, a shared decision was made to commence oral delafloxacin 450 mg twice daily. This approach balanced the risks associated with fluoroquinolone therapy against those of prolonged outpatient IV antibiotic treatment via a PICC line. The patient completed a six-week course of delafloxacin without reported adverse effects. Baseline ECG and repeat after two weeks demonstrated a satisfactory corrected QT interval. Clinical improvement was observed soon after treatment initiation, with complete healing of the fifth metatarsal ulcer by the end of treatment.

**Conclusions:**

Delafloxacin may be an effective option for treating osteomyelitis caused by *P. aeruginosa* where resistance to other fluoroquinolones is present. In this case, its use enabled successful treatment without prolonged IV antibiotic therapy and its associated risks. Shared decision-making between the patient and multidisciplinary team was central to this outcome.

